# Applications of generative adversarial networks in the diagnosis, prognosis, and treatment of ophthalmic diseases

**DOI:** 10.1007/s00417-025-06830-9

**Published:** 2025-04-22

**Authors:** Robert Doorly, Joshua Ong, Ethan Waisberg, Prithul Sarker, Nasif Zaman, Alireza Tavakkoli, Andrew G. Lee

**Affiliations:** 1 University of Cambridge, Cambridge, UK; 2 Department of Ophthalmology and Visual Sciences, University of Michigan Kellogg Eye Center, Ann Arbor, MI USA; 3 Human-Machine Perception Laboratory, Department of Computer Science and Engineering, University of Nevada, Reno, Reno, NV USA; 4 Center for Space Medicine, Baylor College of Medicine, Houston, TX USA; 5 Department of Ophthalmology, Blanton Eye Institute, Houston Methodist Hospital, Houston, TX USA; 6 The Houston Methodist Research Institute, Houston Methodist Hospital, Houston, TX USA; 7 Departments of Ophthalmology, Neurology, and Neurosurgery, Weill Cornell Medicine, New York, NY USA; 8 Department of Ophthalmology, University of Texas Medical Branch, Galveston, TX USA; 9 University of Texas MD Anderson Cancer Center, Houston, TX USA; 10 Texas A&M School of Medicine, Bryan, TX USA; 11 Department of Ophthalmology, The University of Iowa Hospitals and Clinics, Iowa City, Iowa, USA

**Keywords:** Generative AI, Generative Adversarial Networks, Ophthalmology, Machine Learning, Eye Disease, Deep Learning

## Abstract

**Purpose:**

Generative adversarial networks (GANs) are key components of many artificial intelligence (AI) systems that are applied to image-informed bioengineering and medicine. GANs combat key limitations facing deep learning models: small, unbalanced datasets containing few images of severe disease. The predictive capacity of conditional GANs may also be extremely useful in managing disease on an individual basis. This narrative review focusses on the application of GANs in ophthalmology, in order to provide a critical account of the current state and ongoing challenges for healthcare professionals and allied scientists who are interested in this rapidly evolving field.

**Methods:**

We performed a search of studies that apply generative adversarial networks (GANs) in diagnosis, therapy and prognosis of eight eye diseases. These disparate tasks were selected to highlight developments in GAN techniques, differences and common features to aid practitioners and future adopters in the field of ophthalmology.

**Results:**

The studies we identified show that GANs have demonstrated capacity to: generate realistic and useful synthetic images, convert image modality, improve image quality, enhance extraction of relevant features, and provide prognostic predictions based on input images and other relevant data.

**Conclusion:**

The broad range of architectures considered describe how GAN technology is evolving to meet different challenges (including segmentation and multi-modal imaging) that are of particular relevance to ophthalmology. The wide availability of datasets now facilitates the entry of new researchers to the field. However mainstream adoption of GAN technology for clinical use remains contingent on larger public datasets for widespread validation and necessary regulatory oversight.

**Supplementary Information:**

The online version contains supplementary material available at 10.1007/s00417-025-06830-9.

## Key messages

***What is known***

• Deep learning has been shown capable of diagnosis of a range of ophthalmic conditions, but datasets used for training often lack sufficient size, contain variable quality images and are imbalanced.

• Generative adversarial networks (GANs) offer a means to augment data and can enhance diagnosis accuracy through image conversion or resolution improvement.

• GANs moreover offer a predictive capability for disease progression and post-treatment outcomes.

***What is new***

• Recent developments in GANs applied to diagnosis, prognosis and therapy for a range of specific ophthalmic conditions are summarised.

• This review outlines the current state of the art in GAN techniques, facilitating performance comparisons of competing methodologies and points to new approaches.

**Supplementary Information:**

The online version contains supplementary material available at 10.1007/s00417-025-06830-9.

## Introduction

The integration of artificial intelligence (AI) into ophthalmology has the potential to revolutionize ocular disease diagnosis, management, and treatment; to facilitate earlier, more effective interventions; and to enhance patient clinical outcomes. Several characteristics of ophthalmology make it highly suited to AI, particularly the importance of image analysis in the diagnosis and management of ocular diseases [1].

AI is the automation of tasks normally requiring human intelligence, and encompasses machine learning (ML) and deep learning (DL) [2]. ML involves the development of algorithms that learn from experience and improve their performance without requiring specific programming, while DL learns via multi-layer neural networks, allowing for enhanced processing [3, 4]. Several studies have already shown success in training DL models, primarily convolutional neural networks (CNNs), to diagnose conditions, including diabetic retinopathy and age-related macular degeneration, from retinal images with high accuracy and speed [5–8]. The hierarchical structure of CNNs makes them highly suitable for image analysis: convolutional layers apply learnable kernels to the input, pooling layers down sample feature maps produced by the kernels, and fully connected layers map inputs to final outputs [9]. The CNN adapts its features at each level through backpropagation, enabling it to analyse images without requiring pre-processing. However, accuracy and transferability of CNNs depends on the training set size, quality and diversity: image collection may be limited by privacy considerations and by rarity of disease or cost, whilst quality of image may be limited by camera used, and accuracy of labelling [10, 11]. Furthermore disease phenotype (for example retinopathy of prematurity) may vary between populations [11, 12].

Generative AI models synthesise data similar to that on which they have been trained. Generative AI applications in ophthalmology include creating synthetic data to combat lack of real data, improving data quality, enhancing feature extraction and treatment planning. Forms of generative AI include generative adversarial networks (GANs), variational autoencoders (VAEs) and diffusion models (DMs). To illustrate, Fig. 1 from [13] compares the performance of different GAN models used to translate and remove artefacts from fundus images, while Fig. 2 from [14] demonstrates the application of a GAN to augment a database by synthesising images showing various stages of age-related macular degeneration.

**Fig. 1:**
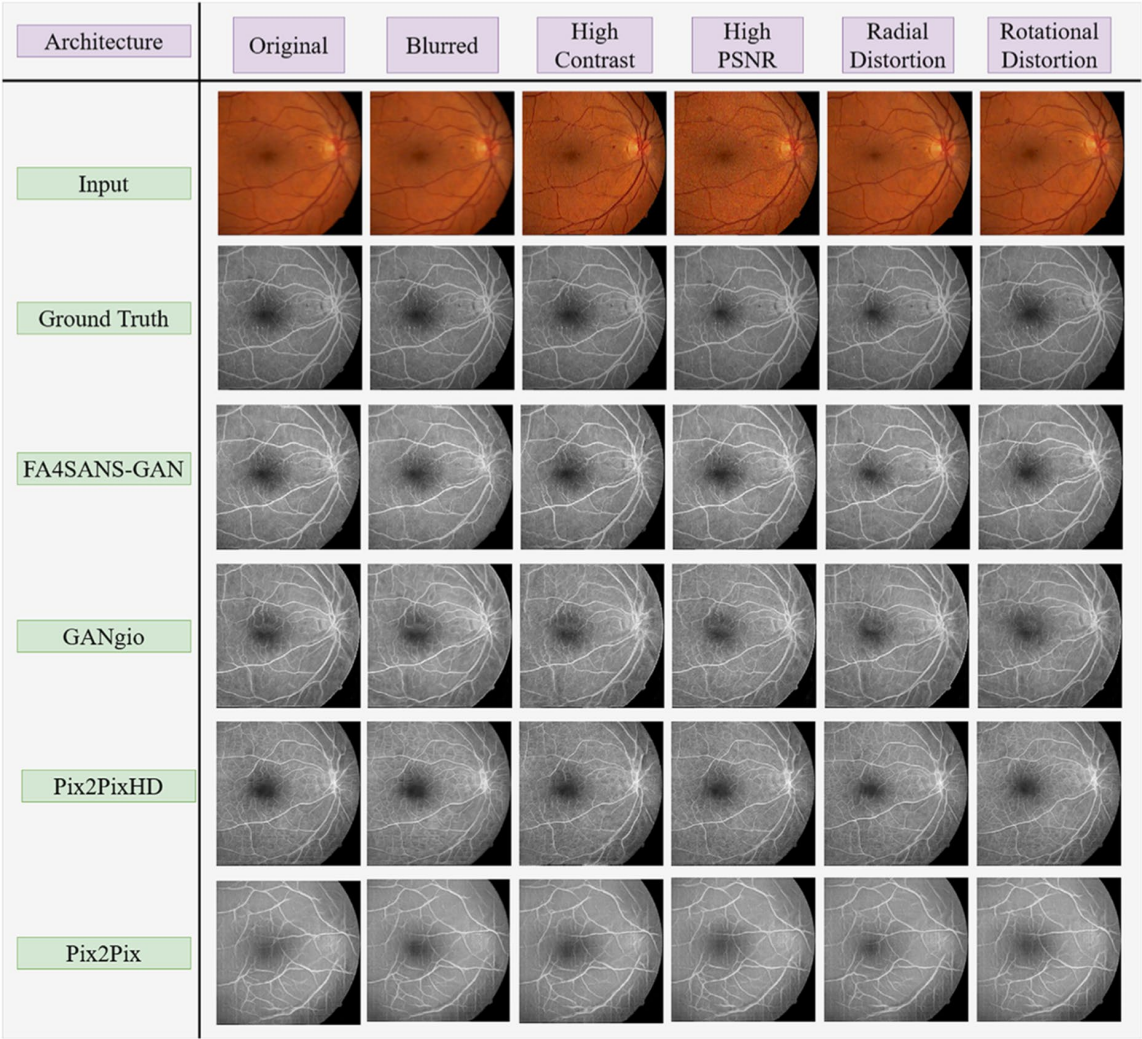
Example of GAN architectures used for image translation and artefact correction. Panel compares performance of different GAN architectures used to generate fluorescein angiograms from normal colour fundus images as well as images showing common artefacts. Note improvement in performance moving from bottom to top row. (PSNR = peak signal to noise ratio). Reproduced from Kamran et al. [13] under CC BY-NC-ND license (http://creativecommons.org/licenses/by-nc-nd/4.0/)

**Fig. 2:**
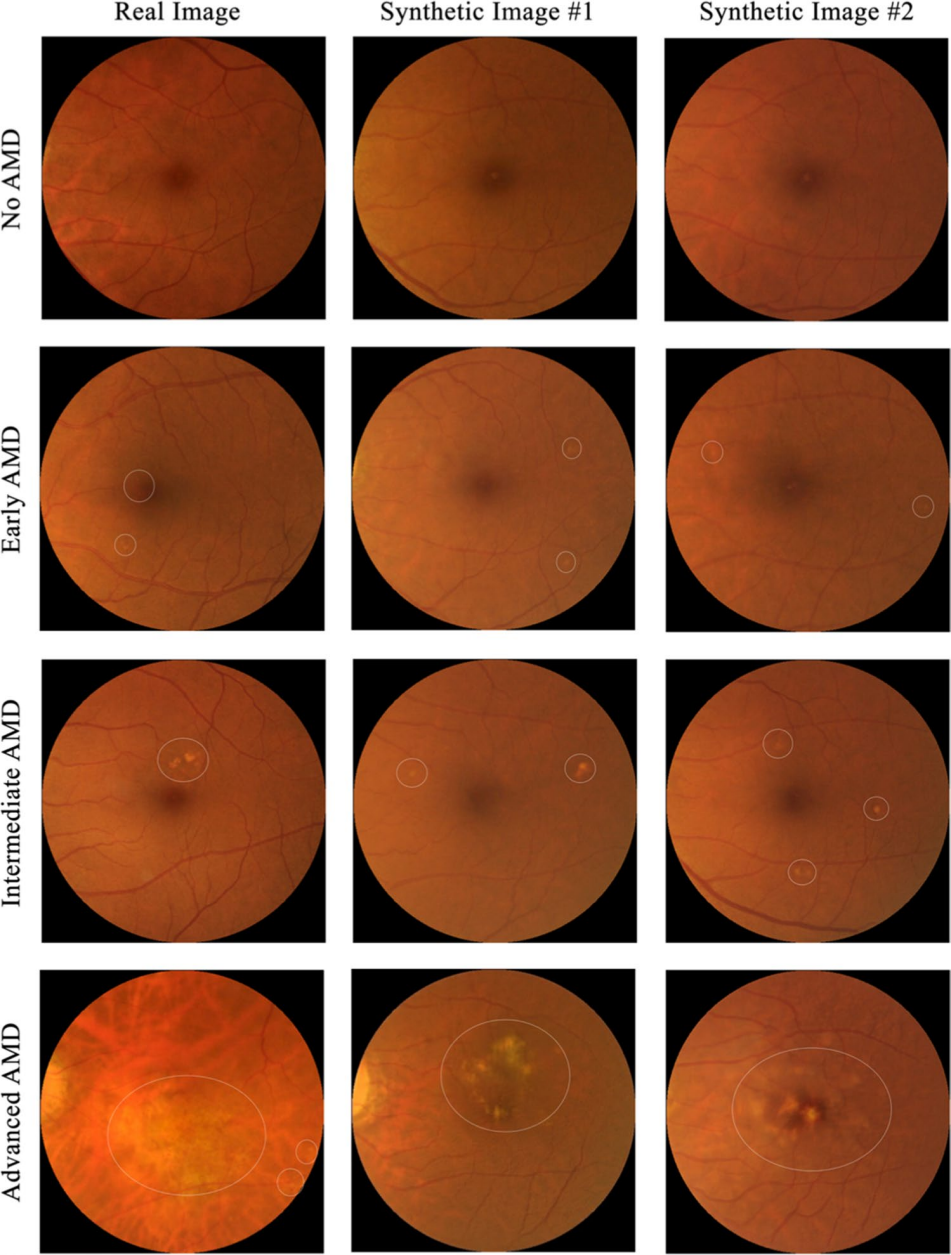
Example of a GAN architecture used to augment a database of normal and AMD fundus images at various stages of disease. First (leftmost) column shows real images and columns 2, 3 show synthetic fundus images. Top row shows no AMD, with progressively more severe AMD moving to bottom row. White circles mark AMD areas on the fundus images. Image reproduced from Wang et al. [14] under CC BY-NC-ND license (http://creativecommons.org/licenses/by-nc-nd/4.0/)

In this paper we review recent developments in the application of generative AI, specifically GANs, to ophthalmic care, covering diagnosis, prognosis and treatment. Our objective is to deepen understanding of how GAN technology may be applied to the field by providing critical condensed commentaries and sources of research findings targeted towards clinical use in diverse applications. We chose eight specific conditions in order to show how GANs are adapted to perform different tasks in ophthalmology, dealing with various imaging modalities to meet specific requirements. The advantage of adopting this broad approach is that it reveals many commonalties both in technical methods and challenges in terms of data and validation, providing a source of information for a range of specialists and developers. Given the emphasis on understanding and breadth of coverage in this work, we adopt the format of narrative review as recommended by Greenhalgh et al. [15], rather than the limitation of a systematic review which is geared towards addressing narrowly focused questions and data summary [15].

We begin with a brief overview of the ophthalmic diseases considered, which cover a varied range of image types. The basic structure of GANs is then outlined before describing methods used for the review. We summarize the review findings and segment generative AI models for diagnosis (Table 1), and prognosis and treatment (Table 2). The supplementary information provides longer summaries of the methods, data used and reported results of papers on diagnosis (Table 3) and on prognosis and treatment (Table 4).

**Table 1:** Applications of Generative Adversarial Networks (GANs) in diagnosis of ophthalmic disease

Disease	Study	Year	Generative aspect	Image type(s)	Datasets
**DR**	Liu J. et al. [16]	2024	Data augmentation	CF	MESSIDOR, DRIVE and RITE
**DR**	Alwakid et al. [17]	2023	Image enhancement	CF	Aptos 2019
**DR**	Shi et al. [18]	2023	Image conversion	CF, FFA	Unpublished + EyePACS and MESSIDOR2
**DR**	Yuan et al. [19]	2023	Improved segmentation	CF	IDRiD dataset
**DR**	Zhou et al. [20]	2022	Data augmentation	CF	Eye-PACS, FGADR
**DR (DME, CNV, drusen)**	Zheng et al. [21]	2022	Semi-supervised learning	OCT	‘Cell’ OCT datasets & trial: ChiCTR1900024528
**DR**	Chen et al. [22]	2021	Data augmentation	CF	EyePACS, FGADR, IDRiD, DRIVE & This study
**DR**	Ju et al. [23]	2021	Image conversion	CF, UWF	Unpublished: data from private hospitals
**DR**	Wang et al. [24]	2021	Image conversion	UWFA	Unpublished
**DR**	Zheng et al. [25]	2018	Augmentation	CF	e_ophtha_EX, DiaReTDB1, HEI-MED, MESSIDOR
**AMD**	Chen et al. [26]	2024	Image conversion	CF, ICGA	Unpublished – on request
**AMD**	Wang et al. [14]	2023	Augmentation	CF	Unpublished from Singapore Integrated DR program
**AMD**	Song et al. [27]	2023	Image conversion	CF, FAF	Unpublished from 2586 patients; LabelMe
**AMD**	Burlina et al. [28]	2019	Augmentation	CF	National Institutes of Health (AREDS) AMD dataset
**ROP**	Hou et al. [29]	2023	Augmentation	CF	Unpublished + DRIVE, STARE, CHASE_DB1 HRF
**Disease**	Study	Year	Generative aspect	Image type(s)	Datasets
**ROP**	Coyner et al. [30]	2022	Augmentation	CF	3477 real images from 534 patients across several US centres; real data unavailable, but provide link to synthetic data
**Multiple, incl. DR, AMD, myopia, cataract, HR** **glaucoma**	Han et al. [31]	2021	Anomaly detection	CF	Unpublished + EyePACS, IDRiD, ODIR- 5 K, Messidor- 2, JSIEC1000
**Glaucoma**	Chaurasia et al. [32]	2024	Data augmentation	CF	20 different databases worldwide; validated on Drishti-GS
**Glaucoma**	He et al. [33]	2023	Image conversion	CF	Unpublished: 843 Topcon-Optain pairs
**Glaucoma**	Sreejith Kumar et al. [34]	2022	Augmentation	OCT	Data from 4 clinical trials & from Singapore Epidemiology of Eye Disease program, Carol Davila Bucharest
**Cataract**	Liu et al. [35]	2024	Image enhancement	CF, UWF	Unpublished from Zhongshan Ophthalmic Centre. 959 CF & 1009 UWF image pairs pre- and post-cataract surgery to train GAN, 100 CFP & 100 UWF pairs for testing
**Cataract**	Luo et al. [36]	2020	Image enhancement (de-haze cataractous images)	CF	Unpublished: 400 clear and 400 real cataract images for training image synthesis and dehaze GANs. 50 pre- and post-surgery images from Wenzhou Eye hospital for evaluation
**Myopia**	Jiang et al. [37]	2019	Improved segmentation	ICGA	Unpublished:76 images (each eye from 38 patients attending Shanghai General Hospital), split into 10,10,9,9 for four-fold cross validation

*CF* colour fundus, *FAF* fundus autofluorescence, *FFA* fundus fluorescein angiography, *IRF* infra−red fundus, *ICGA* indocyanine green angiography, *IOP* intra−ocular pressure, *OCT* optical coherence tomography, *RT* retinal thickness, *SD−OCT* spectral domain OCT, *UWF* ultra−wide fundus, *UWFA* Ultra−wide fluorescence angiography

**Table 2:** Applications of Generative Adversarial Networks (GANs) in prognosis and treatment of ophthalmic diseases

Disease	Study	Year	Generative aspect	Image type(s)	Dataset(s)
DME	Baek et al. [38]	2024	Predict anti-VEGF therapy outcomes (predicted post therapy OCT images)	OCT, IRF	Data from randomized controlled trial (CRTH258B2305, KINGFISHER)
Glaucoma	Hussain et al. [39]	2023	Predict change in visual field at 12 months	OCT	Data from longitudinal study of trabeculectomy patients in Lithuania, OCT, IOP, RT heat map
AMD (nAMD)	Zhao et al. [40]	2024	Predict anti-VEGF therapy effects as above	OCT	Data from GUM (SD-OCT) and from unpublished dataset
AMD (nAMD	Zhang et al. [41]	2023	Predict anti-VEGF therapy outcome	OCT	Unpublished: 46 k paired SD-OCT images from 22 patients
nAMD	Moon et al. [42]	2023	Predict anti-VEGF outcomes for treated with ranibizumab vs aflibercept	OCT	Unpublished images from 842 patients: 419 patients treated with ranibizumab vs 423 with aflibercept
AMD	Pham et al. [43]	2022	Predict drusen development as seen on CF	CF	Unpublished: AMD patients at Kangbuk Samsung Hospital Korea
AMD	Ganjdanesh et al. [44]	2022	Classify current and predict future AMD grade	CF	National Eye Institute AREDS and UK biobank
AMD (nAMD)/	Lee et al. [45]	2021	Predict anti-VEGF therapy after 1 month	OCT, FA, ICGA	Unpublished data from ~ 300 patients treated with ranibizumab or aflibercept at Konkuk University Medical Center, Korea
AMD (nAMD)/	Liu Y. et al. [46]	2020	Predict short term post-anti-VEGF therapy (OCT images)	OCT	Unpublished 526 patients’ data from Peking Union Medical College Hospital
TAO: thyroid-associated ophthalmopathy	Yoo et al. [47]	2020	Predict appearance post orbital decompression for TAO	Facial image	Data in paper supplementary info: pre- and postoperative face photos from patients who underwent orbital decompression
Blepharoptosis	Sun et al. [48]	2022	Predict eyelid contour post-surgery	Facial image	Unpublished: 970 pairs of pre- and post-op images from 362 patients attending oculoplastic clinic Hangzhou, China
Cataract	Frisch et al. [49]	2023	Predict downstream surgical phases, anatomy, and tool use	Surgical videos	50 videos from CATARACTS2020 dataset, of which 25 used for training, 5 for validation and 20 for testing. 101 videos from Cataract101 dataset (70 for training, 20 for validation, 11 for testing)
Myopia	Assaf et al. [50]	2024	Produce synthetic AS-OCT images to help train CNNs to accurately measure vault	AS-OCT images	Unpublished: Post-op OCT scans using MS- 39 platform from participants who underwent EVO or EVO + ICL surgery from STAAR Surgical. 2447 scans from 70 eyes of 51 patients used for training, 2110 from 68 eyes of 52 patients for validation, and 2454 from 88 eyes of 56 patients for testing

### Diseases considered

Ocular diseases represent a significant burden both at individual and population levels globally and even more so in the developing world, where increased incidence of certain diseases (e.g. HIV-related CMV retinitis) as well as reduced access to ophthalmic services [51] make blindness and visual impairment more prevalent [52].

*Glaucoma*, characterised by progressive degeneration of retinal ganglion cells [53], is the leading cause of irreversible blindness worldwide (affecting over 70 million people [54]), but early reduction of intraocular pressure can arrest disease progression [55].

*Diabetic retinopathy* (DR) affects around 1 in 3 diabetics [56], presenting as microvascular lesions that can progress to causing vision loss. Despite the efficacy of various interventions, DR is the leading cause of preventable blindness in people of ‘working age’ [57], often resulting from a delay in diagnosis. DR may progress rapidly, as illustrated by the images of the same eye one year apart in Rom et al. [58]; that image pair also indicates variability in image centering and quality, one of the challenges in automated image classification. DR frequently manifests as *Diabetic Macular Edema* (DME) [59], in which there is retinal thickening in the macular area, caused by leakage of dilated and hyperpermeable capillaries as well as microaneurysms.

*Age-related macular degeneration,* (AMD), is characterised by the appearance of small, yellow deposits in retinal images called drusen bodies, (for illustration, see [60]), which result in progressive degeneration of photoreceptors and retinal pigment epithelium, causing loss of central vision [61]. It is expected to affect ~ 288 million people worldwide by 2040 [62]. AMD can lead to choroidal neovascularization (CNV), in which new blood vessels grow into the sub-retinal space. Kermany et al. [8] compare the appearance of CNV, DME and drusen in optical coherence tomographic (OCT) images, a key modality in guiding therapeutic interventions. To prevent vision loss, enhanced early detection of AMD is vital to reduce the burden of a global aging population [14].

*Thyroid eye disease* is a complex orbital inflammatory disease that most commonly results from hyperthyroidism, causing eyelid retraction, proptosis and impaired eye movement, and can progress to cause blindness [63].

*Retinopathy of prematurity* (ROP) is a retinal vasoproliferative disease that exclusively affects premature babies. In the United States, 68% of infants born below 1251 g in weight developed some form of ROP [64]. A 2010 study estimated that 1700 out of 32,700 babies with any form of ROP would become blind or severely vision impaired [65].

*Blepharoptosis,* defined as the abnormal drooping of the upper eyelid with the eye in primary gaze, may be congenital or acquired [66], and may affect not just superior visual field but overall health-related quality of life [67].

*Cataract* is a partial or total opacification of the crystalline lens [68] causing an estimated 46% of cases of blindness worldwide [69]. It is most commonly age-related [70] and is treated with cataract surgery which typically can restore vision [71].

*Myopia* (short-sightedness) present as images of distant objects coming into focus anterior to the retina, typically resulting from excessive axial eye growth [72]. Although visual acuity in myopia can usually be corrected with glasses, contact lenses or refractive surgery, myopia increases the risk of sight-threatening diseases including glaucoma and retinal detachment [73]. Myopia is estimated to affect 22.9% of the global population, including 2.7% with high myopia (who are at higher risk of side effects) [74].

### Generative artificial intelligence in ophthalmology

Generative adversarial networks (GANs) dominate the literature for generative AI in ophthalmology [75]. GANs comprise separate generator and discriminator networks [76]. The generator produces synthetic images which are input to the discriminator. The discriminator (trained on a dataset of real images) then determines whether the image is real or synthetic. The generator learns from the discriminator’s decisions, adjusting its parameters to produce more authentic-looking images. Generator and discriminator continue to be trained in parallel, maximising the performance of the generator against the discriminator.

**Fig. 3:**
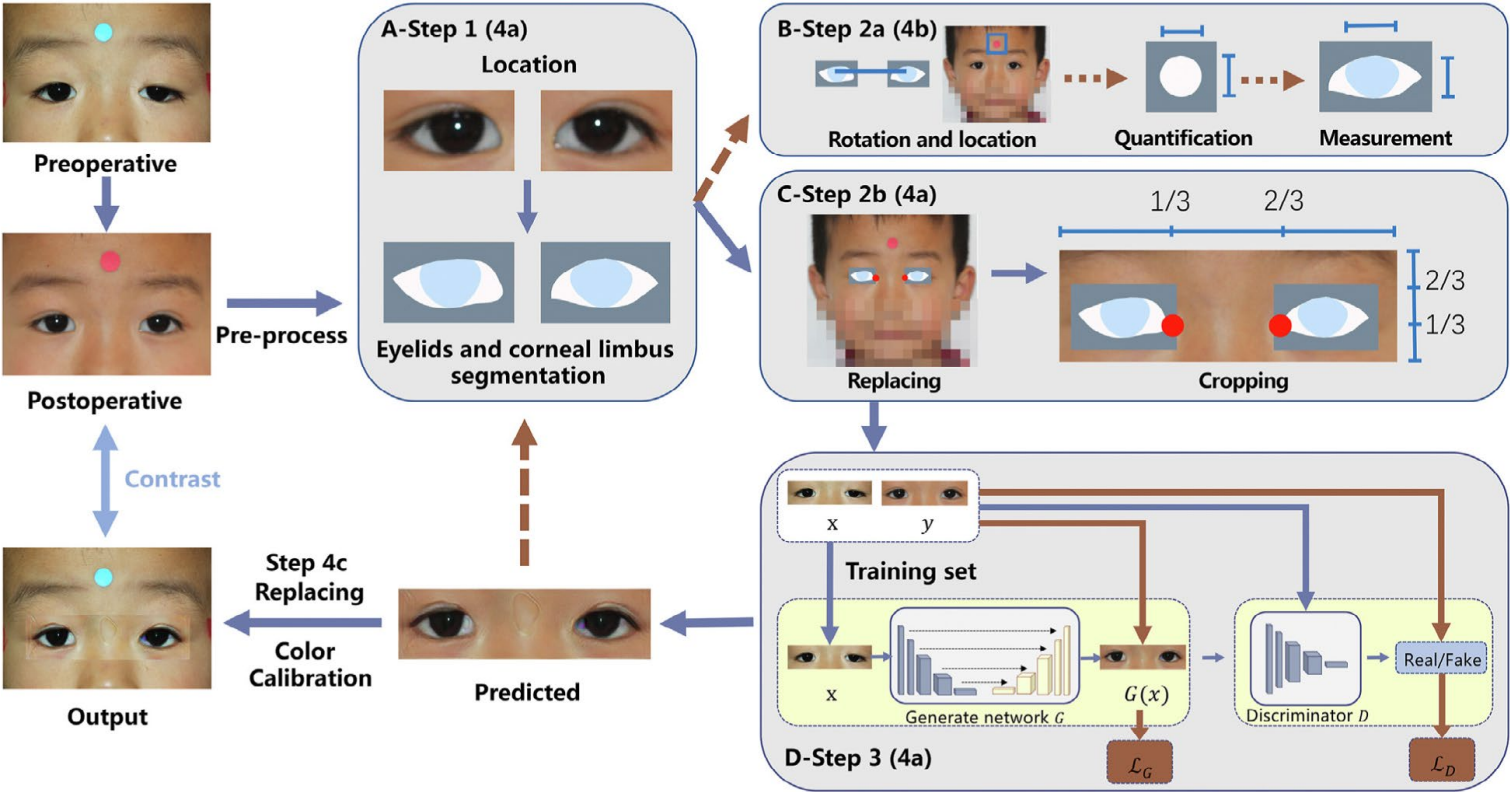
Flowchart illustrating stages in predicting post-operative appearance in blepharoptosis surgery reproduced from [77] under CC BY-NC-ND license (http://creativecommons.org/licenses/by-nc-nd/4.0/). Steps 1 and 2 detail preprocessing steps, with step three illustrating the GAN architecture

**Fig. 4:**
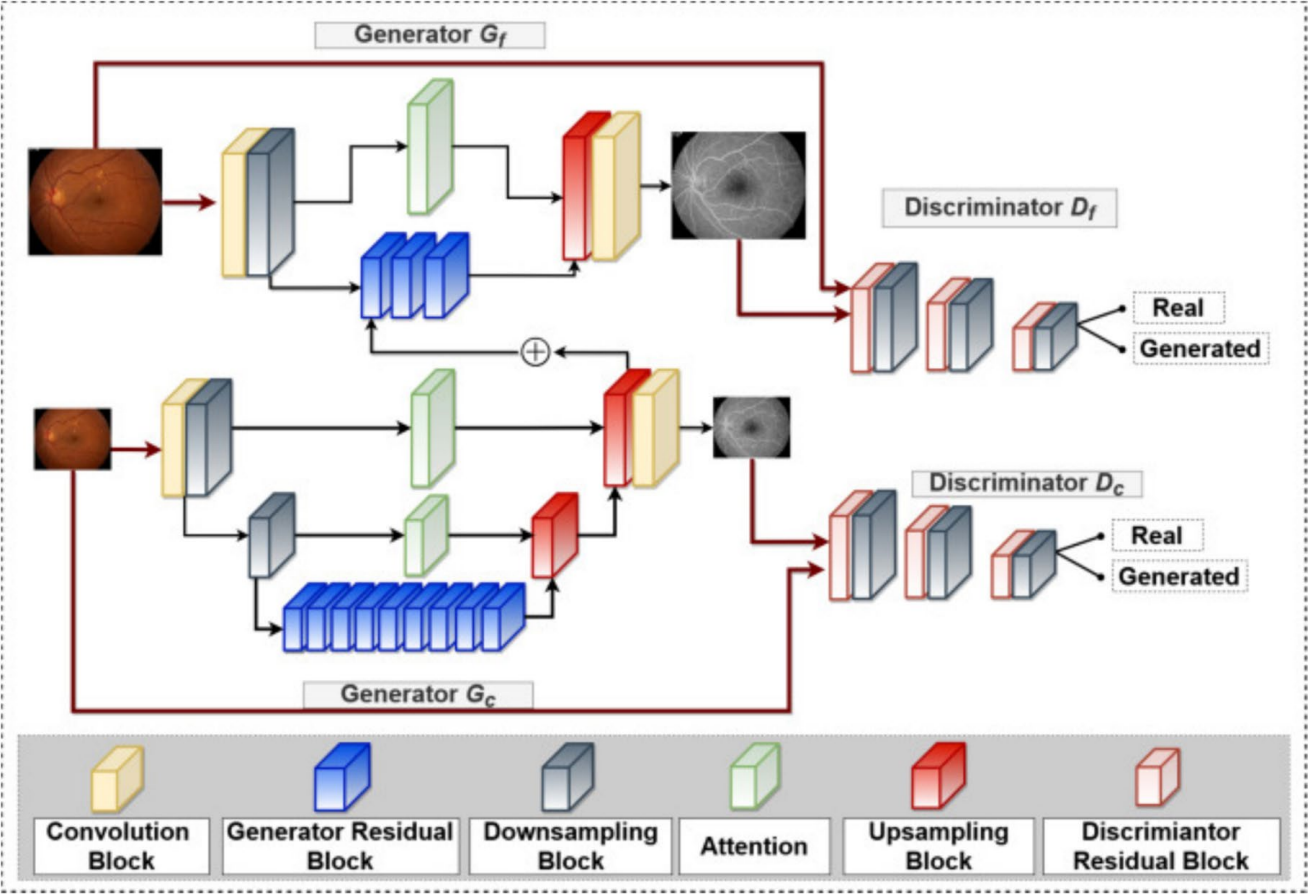
Architecture for FA4SANS-GAN, a fundus image to angiographic image synthesis machine learning framework. This GAN architecture employs two discriminators, two generators, as well as distinct building blocks (convolution, generator residual, discriminator residual, downsampling, upsampling, and attention). The generators employ reconstruction loss while the discriminators employ adversarial loss in this framework to synthesize angiographic data. Reproduced from [13] under Creative Commons License (https://creativecommons.org/licenses/by-nc-nd/4.0/legalcode.en)

Conditional GAN (cGAN) [77] contains a generator which learns to synthesise an output conditioned on an additional input. An example is the Pix2pix GAN, which conditions on input images to generate corresponding output images, as illustrated in Fig. 3 (from [48]), considered further later. In progressively grown GANs (PGGANs or ProGANs) [78], both generator and discriminator gain layers as training progresses, allowing for the development of high-resolution images. Deep convolutional GAN (DCGAN) adds a deconvolutional layer which also enhances image resolution [79]. The CycleGAN architecture contains two generators and two discriminators: it learns to transform input images from the abnormal domain to the normal domain and vice-versa [24]. Figure 4 (from [13]) illustrates the GAN architecture FA4SANS-GAN, utilized to generate angiographic data from fundus images for spaceflight associated neuro-ocular syndrome (SANS), a physiologic barrier to future spaceflight [13, 80]. The architecture comprises two generators, two discriminators, and multiple blocks to generate angiographic images from fundus photos. Yet another GAN architecture, called RegGAN, incorporates a registration network, overcoming the need for pixel-wise alignment of source and target images. Despite the considerable advances in technology that have spawned the different types of GAN, there remain acknowledged deficits in their ability to generate synthetic images of the highest realism. In many cases this is attributed to lack of data. In reviewing GAN approaches, we describe the source and accessibility of data used.

#### Methods

##### Review Methodology

Articles in English catalogued in the PubMed database dated between January 2018 and December 2024. The search was performed using the following search terms: ("generative adversarial network"OR"generative adversarial networks"OR"GAN"OR"deep generative") AND (("diabetic retinopathy"OR"age-related macular degeneration"OR"glaucoma"OR"thyroid eye disease"OR"retinopathy of prematurity"OR"blepharoptosis"OR"myopia"OR"cataract")) generating 328 results. Studies were first selected based on titles and abstracts for relevancy, leaving 75 studies. Works not considered included topics not relevant to this review, non-human studies, studies that did not focus on a specific ophthalmic disease, studies lacking a clear description of the generative model, duplicates or older versions applying less refined methodology to the same problem, and reviews. Based on full text and the above criteria applied again, 42 of the 75 were selected for analysis. The accompanying Tables 1 and 2 (and the concise summaries in the Supplementary Information Tables 3 and 4), highlight key attributes of these works, apart from 6 which provide less detail or are superseded by later studies included in the table.

## Results

### GANs in the diagnosis of ophthalmic conditions

Retinal image datasets used to train ocular disease models often lack sufficient size, contain variable quality images and are imbalanced. Datasets often include few images of severe pathology compared to healthy or mild disease images, thus increasing the risk of model overfitting. In the largest public DR dataset, EyePACS (35,126 training images and 53,576 testing images) 73.67% of images show DR level 0, versus 2.35% and 2.16% for DR levels 3 and 4 respectively [20]. Since collecting data from patients can be time-consuming, costly and must manage privacy concerns, generating synthetic images to augment and balance datasets offers a promising alternative. Additional data augmentation techniques such as transfer learning are also useful to enhance small datasets [81].

In addition to data augmentation, GANs can enhance diagnosis accuracy by image conversion or resolution improvement [82, 83]. Improving the resolution of images input into DL classifiers risks overfitting but can provide more data for training. With image conversion, GAN-generated converted images can be input to the CNN separately from, or alongside the real images, improving classification [13]. Conversion helps standardise images collected in different ways: for example with different cameras/angles/fields of view.

GANs can further improve processing and analysis of real image data by synthesising segmentation results, which identify and extract key features including lesions, so that DL models pay more ‘attention’ to them, [82, 84]. We now discuss uses of generative AI in diagnosis according to ophthalmic disease and according to the major aspect of the work (e.g. image synthesis) in italics. Key details of the cited works are listed in Table 1, with Table 3 in the supplementary information providing condensed summaries of generative aspect, datasets, and reported results.

### Diabetic retinopathy

Since early and targeted treatment can stop DR progressing to irreversible vision impairment, improved diagnosis is essential [85]. Diagnosis of DR typically depends on the presence of lesions including hard exudate, soft exudate, microaneurysms and haemorrhages. These may be scattered and highly similar, limiting CNN success in DR diagnosis [19]. Several studies target GANs to augment training data (specifically of images with lesions) or to enhance feature extraction and lesion recognition.

#### Image synthesis

Chen et al. [22] and Zheng et al. [25] describe GAN-based colour fundus (CF) image synthesis for data augmentation, leading to improved CNN performance on DR classification as detailed in supplementary Table 3. Later, Zhou et al. [20] proposed a high-resolution image generator, incorporating a multi-scale spatial and channel attention generator to improve fine scale feature quality in synthesised CF images for DR grades 0–4. They generated 50,000 images, 10,000 for each severity grade of DR. The accuracy of experts in determining these as synthetic was reported as limited to 65.8%. Adding these 50,000 synthetic images to the CNN training set improved grading accuracy by an average 1.75% across different CNNs (the best performer achieved 89.16%). They also conclude that the generative aspect of the GAN appeared more effective in training set augmentation than traditional methods such as CutMix and MixUp that were incapable of realistic image synthesis. In a different approach, Jeon et al. [86] applied their k- SALSA GAN (based on StyleGAN) to generate synthetic average images based on input features of private datasets. This effectively anonymises data while preserving features, thus providing more data for classifier training. They report high fidelity of generated images, which when used to train a classifier (ResNet18), obtained an accuracy of up 0.705 on EyePACS, nearly comparable to 0.725 for models trained on the original images.

#### Segmentation

Yuan et al. [19] focuses on feature extraction and segmentation targeting DR lesions, achieving high Dice correlation coefficients (overlap between synthetic and real images) of DR lesion maps. Sebastian et al. [87] also achieved high Dice coefficient, pixel-by-pixel accuracy, sensitivity and specificity on three separate datasets using a GAN-based segmentation model. Wang et al. [24] applied a CycleGAN model which learns to convert input images to normal (non-DR) images: subtracting original from generated image identifies possible biomarkers and lesion areas, with CNNs used to analyse the ‘subtraction image’.

#### Translation and Classification

Ju et al. [23] and Shi et al. [18] use GANs to translate regular colour fundus images to alternative modalities which are used to train classifiers: Ju et al. converts regular fundus images into ultra-widefield fundus images, while Shi et al. generates paired fluorescein angiography images. Alwakid et al. [17] reports a significant improvement in DR classification accuracy (98.7% compared to 80.87%) when classifiers were trained on fundus images enhanced with CLAHE (contrast-limited adaptive histogram equalisation) and ESRGAN (enhanced super-resolution GAN). Zheng et al. [21] developed a semi-supervised GAN model (detailed in Table 3) containing a classifier which learned to classify images with diabetic macular edema (DME), drusen and choroidal neovascularisation (associated with DR), achieving greater AUC than a supervised DL classifier when trained only on a small dataset.

Liu et al. [16] introduce further refinements in the generation of synthetic CF images with their VS-GAN architecture. They propose various improvements, first separating out the segmented vessel map (obtained by a classical U-Net architecture), which is fed along with the corresponding training image to the GAN. Furthermore they incorporate a hierarchical variational autoencoder (HVAE) as an image latent encoder. This enables different levels of images to be captured, preserving more style features. Liu et al. report that the addition of synthetic images improved the average accuracy of classification of DR level: using the popular CNN ResNet18, classification accuracy improved from 0.65 (without added synthetic images) to 0.67 (with addition of synthetic images), and using ResNet50 correspondingly from 0.68 to 0.71.

Summarizing results for DR diagnosis, embedding enhancement and segmentation in the process of image synthesis improves quality of images for data augmentation. Data augmentation via GANs improves CNN classification, though by relatively small amounts. Lastly, further development of the k-SALSA or similar approaches may combat the shortage of accessible training data.

### Age-related macular degeneration

#### Image synthesis

Burlina et al. [28] used a PGGAN to synthesise CF images with features of AMD, which retinal specialists had limited ability in distinguishing from real images. A DCNN trained on their synthetic images reportedly had an AUC of 0.9235 for classifying AMD, slightly below that achieved by the DCNN trained on real images (AUC of 0.970). Wang et al. [14] applied the styleGAN- 2 architecture along with a ‘human in the loop’, HITL, to generate synthetic CF images with severe AMD, in order to combat the low proportion of such images in available datasets. They manually selected synthetic images with balanced realness and AMD features from those generated in a first pass of the GAN. These images were then used to augment the data used to train a secondary model. Ophthalmology residents could reportedly discriminate real from synthetic images with accuracy ranging only from 0.54 to 0.64, (similar findings in Burlina et al. [28]). However Wang et al. further report that whilst real and synthetic images appeared equally real for non-referable AMD, their synthetic referable AMD images were more easily identified (accuracy of 0.82). Rather than CF images, Zhao et al. [88] targeted the generation of OCT images (normal, AMD and DME OCT images). They report their custom GAN synthesised images as clearer than those produced by a VAE. Although they found an improvement in classification accuracy, sensitivity, and specificity by 1–2% on the same test set, they conclude that their synthetic images were of lower quality and lacking diversity.

#### ***Translation***

Song et al. [27] used the general purpose pix2pixHD GAN to translate CF images to fundus autofluorescence (FAF) images. They observed that the addition of FAF images to the corresponding CF images improved AMD classification, with the AUC increasing from 0.931 to 0.967. More recently Chen et al. [26] used pix2pixhd to translate CF photos to indocyanine green angiography (ICGA) using GANs on a large dataset (3195 CF images and 53,264 ICGA images from 1172 patients). They found that GANs could be trained to synthesise high-resolution ICGA images based on CF images. As in Song et al., adding generated ICGA on top of CF images reportedly improved AMD classification accuracy. however statistically significant improvement only occurred with addition of early + middle or early, middle and late phase synthesised ICGA images.

Briefly summarising, works incorporating an image translation stage (CF to ICGA) in synthesis report improved classification performance. Secondly, synthetic images of referrable disease are not yet reliably realistic, due to features such as vascular discontinuity, and the blurry texture of choroidal vessels (Wang et al. [14], Chen et al. [26]).

### Glaucoma

Sreejith Kumar et al. [34] used PGGAN models to generate large numbers of synthetic circumpapillary OCT scans, which were graded for authenticity and used to train CNN classifiers. Two experts were effectively unable to distinguish real from fake images (accuracies of 51.8% and 51.3%). The best-performing CNN reportedly achieved higher AUC on both internal and external test sets when trained on the synthetic images compared to the real images (0.97 vs 0.96 and 0.90 vs 0.84 respectively), though real images were noted to be of lower quality He et al. [33] enhanced the generalisability of CNN diagnostic models by using GANs to convert images taken on one type of camera (Optain), to another type (Topcon). The reason given is that the Topcon produces better quality images of the optic disc, which are needed for glaucomatous optic neuropathy (GON) measures such as cup-to-disc area ratio (CDR) and vertical cup-to-disc ratio (VCDR). More recently, Chaurasia et al. [32] pre-processed images of people with and without glaucoma from 20 different databases worldwide in order to create a large uniform set of (17,060) ONH disc images (6874 glaucomatous and 10,186 healthy). These were then applied to train deep convolutional generative adversarial networks (DCGANs), as used previously by Diaz-Pinto et al. [89], to generate ONH disc images for normal and glaucomatous eyes. For performance evaluation, a CNN (vgg19_bn) was trained on two data sets: one with only synthetic images and one with a mix of synthetic and real clinical images, labelled ‘mixed data set’. Chaurasia et al. [32] report their model AUC as attaining 99.85% on internal validation but dropping to 86.45% on external validation. By applying Gradient-weighted Class Activation Mapping (Grad-CAM) they observed that training on synthetic images made the DL less focussed on clinically significant regions, versus when trained on mixed synthetic and real images, where the optic nerve head featured prominently. They conclude that whereas real images are essential in training data, there was no statistically significant difference in classifier performance on unseen data when trained on real or mixed real & synthetic images (accuracy 87.13% vs 86.14%).

To summarise, results for GANs applied to glaucoma show the benefit of image translation (He et al. [33]), demonstrate use of Grad-CAM to probe attention deficits in DL trained on synthetic images and highlight importance of large datasets.

### Retinopathy of prematurity

Hou et al. [29] and Coyner et al. [30] address the imbalance of ROP image datasets by developing GANs to synthesise ROP images, which are used to train CNN classifiers. In Coyner et al. [30], real images were first segmented to form retinal vessel maps (RVMs), which were used to train three PGGANs separately on normal, preplus (mild, but requiring follow up) and plus vasculature (severe vascular abnormalities). ResNet- 18 CNNs trained solely on synthetic RVMs showed significantly higher AUC (0.971) to the CNN trained on real RVMs (0.934) in detecting plus disease.

Hou et al. [29], applied a modified U-Net, U^2^-Net, for RVM generation, and combined an adversarial autoencoder with a custom GAN to generate synthetic CF images. Adding these to the training set of the ResNet34 CNN improved classification accuracy from 73.2% to 91.0%. To summarise, in the case of ROP, when combined with RVM, GANs appear capable of significantly improving classification accuracy. Moreover Coyner et al. [30] also demonstrated using Frechet Inception Distance (FID) and Euclidean distances that synthetic images had significant variety and were distinct from the training set, so are not subject to the same privacy considerations.

### Enhancing cataractous images

Luo et al. [36] developed a two-stage process designed to ‘dehaze’ cataractous retinal scans to improve diagnostic accuracy. To overcome the lack of paired clear and cataractous retinal scans, firstly an unpaired GAN (CataractSimGan) was applied to synthesise cataract-like images from clear retinal scans. In contrast to a typical image-to-image GAN, the output of CataractSimGan need not be similar to the input image beyond the main features (optic disc and blood vessels), which should be the same structurally. Two U-nets were trained to segment blood vessels and the optic disc in the cataractous image. Secondly the synthesised image pairs (clear and cataractous) were input into CataractDehazeNet, a paired GAN based on the pix2pix framework. Testing the performance of CataractDehazeNet on a dataset of fifty cataract images demonstrated improvement in structural similarity measure(SSIM) and peak signal-to-noise ratio (PSNR, an image quality metric) when using images generated by CataractSimGAN (SSIM 0.763, PSNR 20.504) compared with CycleGAN (SSIM 0.701, PSNR 19.053) or purely mathematically simulated (SSIM 0.702, PSNR 15.219). Liu et al. [35] also aimed to enhance both cataractous colour fundus (CF) and ultra-wide field (UWF) images, modifying a CycleGAN structure (called C^2^ycleGAN) for greater focus on critical retinal regions, termed ‘digital ray’. Using pairs of pre- and post-cataract surgery images (either CF or UWF, see Table 3), the GAN learnt to output enhanced images from pre-operative cataractous inputs. The C^2^ycleGAN, reportedly improved similarity between post-surgical real images and synthetic enhanced images (FID scores of 80.78 for CF and 70.64 for UWF) vs using standard CycleGAN (FID scores 102.96 for CF and 90.18 for UWF images). Clinical evaluation of generated images demonstrated improved quality vs non-enhanced pre-op images based on assessment by ophthalmologists at three different levels (resident, senior, expert) and on their diagnostic accuracy of retinopathy and retinopathy subtype detection. For CF images, a greater proportion of synthetic images were graded excellent compared to pre-op images by ophthalmologists at all levels (9.3–15.7% to 17.0–30.7%). Average accuracies of retinopathy detection increased from 78 to 91% in CF images and from 91 to 93% in UWF images. For retinopathy subtype detection, the ophthalmologists achieved higher accuracy for DR and pathological myopia detection, and sensitivity for AMD in CF images, and accuracy for retinal detachment in UWF images when using synthetic images; (further results in Table 3). In summary, GANs have been shown to be well adapted to the task of revealing retinal pathologies obscured by cataract. Assessment of GAN performance through the involvement of ophthalmologists at different experience grades, as in Liu et al. [35] is essential for verification and ideally should be included in all further studies..

### Myopia

Detection of linear lesions (including lacquer cracks and myopic stretch lines) in myopic patients identifies those at particularly high risk of visual impairment [90]. Jiang et al. [37] developed an improved conditional GAN for automatic segmentation of linear lesions in ICGA retinal images. Although ICGA images visualise linear lesions well, segmentation is limited by their complex shape, which often mirror the structures and gray levels of retinal vessels. Training the GAN using four-fold cross validation achieved a Dice Similarity Coefficient of 69.21%, accuracy of 98.92%, sensitivity of 77.63% and specificity of 99.29%. Intersection over union ratio (IoU) however was only 52.95%, with the authors reporting the small size of linear lesions means that minor errors in the results can dramatically reduce IoU. In summary, the dataset only contained 152 images (two images at two different times from each eye of 38 patients), which is insufficient. Enlarging the enlarging the dataset should improve segmentation performance and increase confidence level in the results.

### Multiple diseases

A single diagnostic algorithm to screen for multiple diseases based on fundus imaging would revolutionise ophthalmic care. Han et al. [30] developed a GAN similar to an automatic encoder: the model is first trained on normal samples then ‘tuned’ on a set containing normal and abnormal samples. The GAN develops a latent vector space (a sort of compressed feature map) by minimising several loss functions, via calculating anomaly scores for each input image in the test set. On the external test set, the network achieved an overall diagnostic accuracy of 80.39% (split into 83.89% for glaucoma, 83.44% for cataract, 78.37% for AMD, 82.37% for hypertensive retinopathy and 88.78% for myopia). In summary, a single diagnostic algorithm is clearly highly attractive. However given that GANs targeted towards specific pathologies achieve higher accuracies, it may be that GANs trained in this way may be most useful as a first stage screening tool.

## GANs in prognosis and treatment

Pharmaceutical and surgical interventions can vary in efficacy and side-effects between individuals, thus individualised risk prediction would assist both patients and clinicians. Accuracy of prediction is commonly determined by inputting a pre-treatment image into the GAN and comparing the synthetic image produced to a ground-truth future image. This problem is well suited to conditional GANs, with the generator learning to synthesise a future image conditioned on the input image and timescale. Table 2 and supplementary Table 4 summarise the basis and results of works in which generative AI is targeted towards treatment and prognosis of the considered pathologies.

### Diabetic macular edema

Injection of anti-vascular endothelial growth factor (anti-VEGF) represents the primary treatment option for DME [38]. However clinical trials suggest that approximately 20% to 40% of eyes with DME are refractory to monthly intravitreal VEGF monotherapy. The use of OCT image-derived measures (intraretinal cystoid fluid, total retinal thickness (RT), outer nuclear layer, subretinal fluid (SRF), and hyper-reflective foci (HF)) have emerged as likelihood indicators for suboptimal treatment response to anti-VEGF therapy in DME eyes [91]. In a comprehensive study of methods, Baek et al. [38] compared the performance of the GAN models CycleGAN, UNIT and regGAN in generating predictive OCT B-scan images following long-term anti-VEGF treatment. Model inputs comprised either just the baseline B-scans or these were combined with additional OCT, thickness map, or CF images; the generated B-scan images were then compared with actual images at week 52. It was found that the addition of CF and RT heatmaps did not improve performance, ascribed to their different character compared to OCT images. However, incorporating OCT scan data from weeks 4 and 12 significantly improved performance, with positive-predictive- and negative-predictive-values (PPV and NPV), sensitivity and specificity values ranging above 0.9 and reaching 1.0 for CycleGAN and regGAN when trained on OCT images from weeks 0,4, and 12 weeks. Important conclusions from this work are firstly that the incorporation of longitudinal image data significantly improves outcome prediction. Secondly, the ability to generate realistic outcome images could be a useful aid in educating patients with DME, and thus improving patient compliance and disease management.

### Age-related macular degeneration

Age-related macular degeneration (AMD) progresses through three stages: early, intermediate and advanced [44]. Early and intermediate AMD are typically asymptomatic, reinforcing the importance of early screening and prognostic models [44]. Advanced AMD can occur in wet or dry forms (or a combination of both) [92]. Wet AMD is characterised by choroidal neovascularisation (CNV); photoreceptor cells and the outer retina are damaged by blood vessel growth [93]. Dry AMD is characterised by accumulation of drusen along the basal surface of the retinal pigment epithelium, leading to photoreceptor damage [94]. Ganjdanesh et al. [44] and Pham et al. [43] both develop cGAN models to generate future CF images. Pham et al. [43] uses a multi-scale deep learning model to extract drusen segmentation information from images, then inputs fundus images, drusen masks and time period to a GAN model. Results are quantified by classifying drusen size of predicted and real image: an average accuracy of 55% was achieved over up to 5 years after baseline. Ganjdanesh et al. [44] input synthesised images into a CNN classifier which scored the image as either advanced AMD or non-advanced, achieving accuracy of 0.73–0.77 over 4 years post baseline. Several studies predict the post-treatment status of AMD, focusing on the response to anti-VEGF and making use of OCT images, which provide detail and at depth. Choroidal neovascularization, CNV, primarily results from elevated VEGF level [95], with anti-VEGF the first-line therapy [96], although individual responses are highly variable [97]. Lee et al. [45] trained GANs on OCT images alongside FA and ICGA images taken at baseline and 1 month after final anti-VEGF injection. From a test set of 150 baseline OCT images, 135 of the predicted (i.e. synthesised images) were of acceptable quality for grading. Two ophthalmologists assessed both real and synthetic images and analysed the presence of four different types of lesions. Although prediction accuracy, sensitivity and specificity were reported as generally better than achieved by conventional CNN model, the sensitivity was low. Liu et al. [46] developed a GAN (based on pix2pixHD) that predicted post-treatment macular status (wet/dry) based on pre-VEGF images, achieving an accuracy of 0.85. Zhang et al. [46] extended the Pix2pix model, creating a Single-horizon Evolution Network to predict SD-OCT images post-anti-VEGF therapy. PSNR, SSIM and learned perceptual image patch similarity were reported as better than achieved with competing methods.

Moon et al. [42] investigated GANs use for short-term anatomical outcome prediction in neovascular AMD. They focussed on whether the network could predict differences in anatomical outcomes (as seen in OCT images) between two anti-VEGF treatments, ranibizumab vs aflibercept. In their study, an Attention-GAN was trained separately using data from approximately 400 patients in each treatment category and tested using data from 49 patients in each category. Their model reportedly scored overall higher sensitivity than two experts in predicting the presence of residual retinal fluid after treatment, but with lower specificity and broadly comparable accuracy. Zhao et al. [40] developed a biomarkers aware GAN to synthesise post-anti-VEGF OCT images, using images from large datasets (GUM and locally collected). Clinicians (3) judged 95.5% of the synthetic images as realistically detailed, while comparing presence/absence of four relevant biomarkers in synthetic images achieved accuracies between 81.0% and 92.1%. Success of anti-VEGF treatment was defined as reduced retinal fluid and/or subretinal hyperreflective material and pigment epithelial detachment: predictive power accuracy of the synthetic images was 95.2% at 1 month post treatment, 95.2% at 3 months and 85.7% at 12 months. In summary, incorporation of biomarker attention features and possibly multi-modalities appear to offer the most promising avenues for further developments in this area.

### Glaucoma

Although glaucoma tends to be slowly-progressing and effective treatments exist for reducing intraocular pressure, patients in which it progresses rapidly are at high risk of severe vision loss [98]. Hussain et al. [39] used the pix2pix GAN framework to synthesise OCT-B scans at future time points which were input alongside other modality data to a CNN classifier to predict vision loss at 6,9 and 12 months post-baseline. Training the CNN on real images and data until M6 (6 months) achieved an AUC of 0.82 for 12-month visual field change prediction. Adding synthetic M9 and M12 images to the training data increased AUC marginally but not with statistical significance. However, repeating this at M3, adding synthetic M6, M9 and M12 images to the real data, the AUC for predicting visual field change at 12 months improved from 0.76 (real data) to 0.81, with *p* = 0.038). However the dataset used for training and evaluation contained only 100 patients of a single ethnicity, all of whom were undergoing trabeculectomy for surgical lowering of IOP, therefore generalisability of the results is limited.

### Cataract

Cataract surgery is one of the most commonly performed procedures worldwide (4000–10,000 operations per million people worldwide) [99], and is highly successful (up to 96% of patients had improved visual acuity 4 months post-surgery) [100], however publicly available video recordings of the surgical procedures are scarce. Frisch et al. [49] developed a motion-translation model (MT-UNIT) to generate predictive images showing downstream data including tool use classification, surgical phase prediction and anatomy and tool segmentation. Image-to-image translation models are limited in their application to sequence-to-sequence translation, generating artifacts of temporal inconsistency. The model developed in the study uses a variational autoencoder structure to generate a target domain and a pre-trained Recurrent All-Pairs Field Transform (princeton-vl/RAFT) model to extract the optical flow from the sequence and ensure temporal consistency. Performance of the model was assessed by comparing temporal consistency, image translation quality and downstream task performance against other models (CycleGAN, RecycleGAN, UNIT and OF-UNIT). The MT-UNIT model consistently performed well, scoring the best SSIM, difference in optical flow fields, feature distance across sequences and warping errors out of all models. The model consistently scored at least second for Frechet Inception Distance, Kernel Inception Distance and diversity of translated images, and scored better in performance evaluation for downstream surgical phase classification (F1 score of 0.296 and average precision of 0.297 vs 0.285 and 0.265 for OF-UNIT, the next best scoring model). However many failure cases still occur, and the authors aim to control this by developing a tighter structure across the shared latent space between domains.

### Myopia

Myopia can be treated with the insertion of a phakic intraocular lens (IOL) between the cornea and crystalline lenses [101]. Posterior chamber phakic IOLs must be carefully placed during surgery in order to achieve an optimal vault (distance between posterior surface of the ICL and anterior surface of the crystalline lens). High vaults (> 750 µm) can increase risk of angle-closure glaucoma, while low vaults (< 250 µm) may lead to cataract formation [102]. Vault measurement requires an expert operator to manually place calipers on OCT images, and automation of measurement by CNN models is limited by the lack of diversity of vault measurements in available images [102]. Assaf et al. [102] applied a GAN, (SWAGAN, described in Assaf et al. [50]), previously trained on OCT images to generate anterior segment OCT (AS-OCT) images, which were each then further processed by image editing to generate a set of images with varying vault measurements. The performance of a CNN in vault measurement was compared to manual measurement by experts when trained on authentic data only (2447 AS-OCT B-scans from 70 eyes of 51 patients) or authentic data and GAN data combined, with or without image editing. Training on authentic data only achieved a mean average error (MAE) of 44.68 µm, improving to 24.89 µm when authentic data was combined with vault resizing image editing. Training on authentic data augmented with GAN data achieved an MAE of 35.10 µm, improved to 24.83 µm when combined with vault resizing, nearly reaching the approximate error margin of approximately 18 mm in manual caliper measurements reported in Dhaini et al. [103] (± 5 pixels with axial resolution of 3.6 µm on MS39 OCT). Although addition of GAN data improved CNN performance, the relative effect was much lower than that of image editing software. The authors also reported a relatively higher error rate when detecting vaults < 100 µm, limited by the small number within the training set.

### Thyroid eye disease

Although thyroid eye disease is typically treated medically [104], in some cases orbital decompression surgery is required to prevent optic neuropathy and reduce exophthalmos. Decompression surgery is neither effective for all patients nor without risks, with possible complications including postoperative numbness and new postoperative diplopia [105]. Yoo et al. [47] applied a conditional GAN model to generate synthetic post-op images, which were compared to real post-op images using SSIM and mean absolute error (MAE) metrics. Synthetic images generated by the cGAN model achieved better SSIM than those generated by the Pix2pix and CycleGAN models (MAE stayed similar). A CNN classifier achieved significantly better AUC (0.957) on the test set of images when trained on an augmented dataset of 76 real pairs of images and 500 synthesised by the GAN, compared to when trained only on the 76 real images (0.824) or trained on the 76 real images with simple augmentation methods (0.872).

### Blepharoptosis

Blepharoptosis is one of the most common disorders of the upper eyelid, and can affect appearance and vision. Surgery can be highly effective [66] however without a reference for expected cosmetic surgical outcome many suffer from pre-operative anxiety [106]. Sun et al. [48] trained the Pix2pix cGAN structure to generate post-surgery eyelid structure predictions. Accuracy was quantified using the ‘overlap ratio’: the ratio of the intersecting area to the union area between the predicted and ground truth post-op images. Mean overlap ratio was 0.858, and multiple radial mid-pupil lid distances (eyelid contour analysis) showed no significant differences between synthetic and real post-op eyelids.

## Conclusion

Collectively, the works incorporated in this review describe the current state of the art in GAN techniques for clinicians and vision scientists, providing performance comparisons of the different methodologies used. The underpinning GAN techniques outlined in the text and supplementary tables highlight both current limitations and emerging new developments, providing guidance for vision researchers to build on existing knowledge.

From the variety of applications: creation of synthetic data to combat the lack of real data, improving data quality, enhancing feature extraction and treatment planning, GANs clearly offer great potential to improve ophthalmic diagnosis and treatment. Furthermore, the predictive capabilities of conditional GAN models also may provide patients and clinicians with accurate disease progression and post-treatment outcome information, allowing for better-informed decision-making and patient education.

Despite the considerable advances in technology that have spawned the different types of GAN noted above, there are acknowledged deficits in their ability to generate synthetic images of the highest realism, which in many cases is attributed to lack of data. For example, as discussed real and generated ICGA images could be discerned based on image style alone, and nearly all the generated ICGA images could be distinguished from the authentic ones, [26].

Currently datasets sampling single populations typically lack diversity in participants and image characteristics (camera quality, focus of image among others), limiting the variance of GAN-synthesised images. Larger datasets – specifically including more cases of advanced disease – will undoubtedly assist in improving the realism of generated images and thereby disease classification, staging and prognosis. Furthermore, the lack of diversity of image datasets should be considered when interpreting performance measures of the GANs; the test datasets may not be representative of the general population so significant regulation will be required prior to clinical implementation. As mentioned in Assaf et al. [102], significant improvements in CNN performance reported in the literature when it is trained using a combined real and GAN-generated image dataset versus real data alone can be misleading; if the GAN is trained on a distinct dataset then additional ‘training data’ is used indirectly, and the relative contribution of the GAN-generated images is overestimated.

Aside from the need for more and better data, this review shows that GAN techniques continue to evolve. A recent development is to embed biophysics knowledge by incorporating laws of physics or known physiological constraints within the GAN, as recently proposed [107, 108]. In [107], mathematical models were used to generate synthetic retinal vasculature networks that follow established physiological scaling and flow physics (Murray’s law and Poiseuille flow). These were then processed to match clinical OCT-A data and used with three CycleGANs, so as to translate synthetic images to correspondingly three different target styles (retinal photographs, OCT-A or fluorescein angiography, FA). Moreover the CycleGAN-facilitated detailed vasculature segmentation provided an input for simulations of blood flow, potentially providing further insights into ophthalmic pathologies.

With greater access and improved techniques, we expect performance to improve further in the near-term facilitating validation, adoption and standardisation of the technique. The increasing availability of ophthalmic databases and the trend for many researchers to make complete codes available online has made it easier for new researchers to enter the field, promoting both open-source software and should facilitate more widespread adoption and validation of GANs in ophthalmology.

Drawing together the results presented by the various studies, we conclude that the lack of openly accessible data of sufficient size coupled with independent reproduction and evaluation of claimed results presents the major obstacle to widespread clinical adoption of generative adversarial networks in particular, and more widely to generative AI. There is also the challenge of multiple factors in pathologies. So whilst we report that in prognosis, realistic OCT images post anti-VEGF therapy have been generated, there are multiple prognostic factors at play. GANs are thus far from replacing clinical decision making, but may nevertheless be used to assist clinical practice, not least for patient education.

In this review we outline the GAN approaches associated with specific diseases, highlighting methods and key results. The primary purpose is to aid both clinicians wishing to become familiar with applications of this technology and vision scientists who seek an overview of ophthalmic applications of GANs. Given the ubiquitous nature in the broad field of biomedical engineering of tasks such as image segmentation, incorporation of multi-modal data and image-based prediction, this review should also be of use to researchers wishing to develop applications for ophthalmic health care.

## Supplementary Information

Below is the link to the electronic supplementary material.

Supplementary file1 (DOCX 68 KB)

## Data Availability

No data used in this review.
