# Supplementary material for: Applications of generative adversarial networks in the diagnosis, prognosis, and treatment of ophthalmic diseases
Source: Graefes Arch Clin Exp Ophthalmol. 2025 Apr 22;263(8):2117–34. doi: 10.1007/s00417-025-06830-9 (PMC12414077; doi:10.1007/s00417-025-06830-9)
Supplement: Supplementary file 1 — Supplementary file1 (DOCX 68 KB) [file 417_2025_6830_MOESM1_ESM.docx]

**Supplementary material**

**Table 3** Summaries of papers describing applications of GANs in diagnosis of ophthalmic disease

| Disease/  Study | Year | Generative  aspect | Method | Datasets | Reported Results |
| --- | --- | --- | --- | --- | --- |
| DR /  Liu J. et al.  [63] | 2024 | Data augmentation | cGAN type: Vessel and Style Guided GAN (VSG-GAN), to process color fundus (CF) images. UNet vessel segmentation & hierarchical variational autoencoder (HVAE) to capture different image detail levels from latent space. | MESSIDOR, DRIVE and RITE | Improved performance on image synthesis. Adding synthetic images only marginally improved DR classification level accuracy by ResNet18 (0.65 to 0.67) and ResNet50 (0.68 to 0.71) |
| DR/  Alwakid et al. [61] | 2023 | Image enhancement | Contrast limited adaptive histogram equalisation filter (CLAHE) + enhanced super-resolution GAN (ESRGAN). | Aptos 2019: 3662 retinal images total, with 1805,370,999,193,295 graded respectively: DR absent, mild, moderate, severe, proliferate | Results with enhancement superior to results achieved on APTOS dataset with other methods. |
| DR/  Shi et al. [60] | 2023 | Image conversion | CF photos and paired fundus fluorescein angiography (FFA) images input to pix2pixHD GAN generates FFA images from CF photos. Swin-transformer model to classify images based on CF alone or CF + generated FFA | CF and FFA images augmented with crops, flips, rotations to give 51,370 venous CF-FFA pairs and 14,644 late-phase pairs from 4438 patients. EyePACS and MESSIDOR2 to validate. | Use of generated FFA images alongside CF images in Swin-transformer training improved classification accuracy and AUC. MESSIDOR2 accuracy up from 0.791 for CF to 0.870 for CF + venous + late FFA. |
| DR/  Yuan et al. [54] | 2023 | Improved segmentation | ‘GAU-net’: Res-U net with FESA (feature extraction of self-attention) generator and PatchGAN discriminator. CF images input to the generative network which synthesises segmentation results. | IDRiD dataset (81 images) + traditional data augmentation methods | Achieved better Dice correlation coefficients (overlap between synthetic & real images) of 75.7%, 76.53%, 50.06%, 45.89% for hard exudates, soft exudates, micro aneurysms, haemorrhages |
| DR/  Zhou et al. [48] | 2022 | Data augmentation | ‘DR-GAN’: custom GAN generator with spatial and channel attention module to generate synthetic CF images for DR grade 0-4. CNN diagnosis accuracy compared for CNN models trained on real vs augmented dataset. | Eye-PACS (35126 training images and 53576 testing images), FGADR (1842 images with pixel level annotations to learn structural and lesion masks). | Grading accuracy of several CNNs improved by addition of GAN synthesised images at different DR grades. (Grading accuracy on EyePACS up by 1.75% (average) and kappa by 1.87%) |
| Table 3 (continued) | | | | | |
| Disease/  Study | **Year** | **Generative**  **aspect** | **Method** | **Datasets** | **Reported Results** |
| DR (DME, CNV, drusen)/    Zheng et al. [62] | 2022 | Semi-supervised learning | Semi-supervised GANs with two discriminators: first (unsupervised) determines if OCT image is real or synthetic, second (supervised) predicts image class. For training small labelled dataset combined with large unlabelled dataset. | Extracts from ‘Cell’ OCT datasets[8]: small labelled for supervised training; (for CNV, DME, drusen and normal); unlabelled for unsupervised training. Validation data: ChiCTR1900024528 | Semi-supervised GAN trained with small labelled dataset had similar average performance as DL model trained in labelled Cell dataset, AUC (0.98). But generated image quality less than ProGAN  . |
| DR/  Chen et al.[55] | 2021 | Data augmentation | Two generative models RFGAN1 and RF-GAN2 synthesise 50,000 fundus images (10,000 of each grade). Compare CNN performance when trained on real or augmented datasets | EyePACS, FGADR, IDRiD and DRIVE (two small datasets with pixel-level annotations), private dataset (2738 images | Training on augmented dataset improved average accuracy and kappa across the CNNs by 1.63% and 1.82% respectively (EyePACS); by 1.24% and 1.35% FGADR; private dataset, by 1.04%, 1.13%. |
| DR/  Ju et al.[59] | 2021 | Image conversion | Modified CycleGAN model with a consistency regularisation term applied to convert regular fundus images to ultra-widefield fundus (UWF) images, and to enhance the number of UWF images for training DL classifiers. | Regular fundus images and UWF fundus images acquired from private hospitals and annotated by experts. Total of 669 regular images and 178 UWF images. | UWF-trained model scored higher accuracy vs regular fundus image-trained model & improved by adding synthetic images; but still worse than classifying on original regular fundus images |
| DR/  Wang et al. [46] | 2021 | Image conversion | CycleGAN network: 2 generators and 2 discriminators: transforms input images from abnormal domain (DR) to normal domain (no DR) and vice-versa. Subtracting original image from generated image identifies possible biomarkers and lesion areas for CNN classifiers to determine DR type | 399 Ultra-wide fluorescence angiography (UWFA) images from 280 DR patients and 119 normal patients. Traditional flip, rotation and translation augmentation methods applied. | Performance evaluated on original UWFA images, compared to results from images with the mask of the 7-standard field. UWFA classification results: 100% accuracy for normal, 94.50% for PDR, 76.61% for NPDR. 7-SF results: 94.12% accuracy for normal, 80.73% for PDR, 54.97% for NPDR  . |
| DR/  Zheng et al. [56] | 2018 | Augmentation | pix2pix-based architecture used to generate 120 synthetic images, of which 86 images with no artifacts selected. An ‘MU-net’ classifier trained on the 60 real images either with or without the 86 synthetic images (MU-net or GAN+MU-net) and is diagnostic performance (identifying exudates) was compared. | 60 images from e_ophtha_EX dataset (82 images, of which 47 contain exudates) used to train GAN model. Classifier (MU-net or GAN + MU-net) accuracy compared across different datasets: e_ophtha_EX (22 images), DiaReTDB1, HEI-MED MESSIDOR. | Addition of cGAN-synthesised images to improve MU-net performance in exudates segmentation. Achieved higher F1 score on all datasets (92.79% vs 92.66%, 92.46% vs 91.41%, 91.27% vs 90.72% and 94.34% vs 90.58% for e_ophtha_EX, DiaRetDB1, HEI-MED and MESSIDOR respectively). |
| Table 3 (continued) | | | | | |
| Disease/  Study | **Year** | **Generative**  **aspect** | **Method** | **Datasets** | **Reported Results** |
| AMD/  Chen et al. [67] | 2024 | Image  conversion | GAN (pix2pixHD) used to translate CF photos to indocyanine green angiography (ICGA) | Dataset not open – on request. 3552 CF images and 228,000 ICGA images. sourced from 3094 patients who had been diagnosed with a range of ocular diseases, including age-related macular degeneration (AMD) | Adding generated ICGA images improved AMD classification accuracy compared to CF alone, AUC improved from .9312 to 0.9688 using all phases of ICGA; use of just early-phase ICGA did not significantly improve performance. |
| AMD/  Wang et al. [14] | 2023 | Augmentation | PGGANs trained on AREDS AMD fundus images to generate synthetic images - latter evaluated by two experts. Deep CNNs trained on either synthetic images (DCNN-S) or real images from AREDS (DCNN-R). Human-in-the-loop model (HITL) to enhance StyleGAN-2 generation of images with severe AMD | 125,012 macula-centred fundus images from Singapore Integrated DR program: 80% for training, 20% for validation of GAN model. There were only 67 severe AMD images in the original training dataset. | Training of GANs with sequentially adjusted train data delivered greater numbers of severe AMD (500+). Average accuracy of 4 ophthalmology residents in distinguishing real from fake was 0.79 |
| AMD/  Song et al.  [66] | 2023 | Image  conversion | 6790 color fundus – fundus autofluorescence (CF-FAF) image pairs from 1850 participants used to develop matching synthetic FAF images from CF data. (GAN based on pix2pixHD) | 7049 (CF) images + 8410 fundus autofluorescence (FAF) images from 2586 patients; LabelMe dataset for testing | Testing on LabelMe dataset showed quantitative outcomes of AMD classification significantly better with inclusion of generated FAF images |
| AMD/  Burlina et al. [64] | 2019 | Augmentation | PGGANs trained on AREDS AMD fundus images, generating synthetic images. Synthetic images evaluated by two retinal experts. Deep CNNs trained on either synthetic images (DCNN-S) or real images from AREDS set (DCNN-R). | National Institutes of Health Age-Related Eye Disease Study (AREDS) AMD dataset (133,821 images) | Experts had difficulty distinguishing real and synthetic images: accuracy ~60% (expert 1), ~54% (expert 2). DCNN-S performed only slightly worse than DCNN-R, accuracy of 82.92%, AUC 0.9235 vs 91.12% and 0.9706 for DCNN-R. |
| ROP/  Hou et al.  [72] | 2023 | Augmentation | Image module for segmentation generates retinal vascular network maps, which are input into autoencoder module which generates synthetic vascular networks. GAN-based model generates synthetic fundus images based on the input of synthetic vascular networks | Guangdong Hospital-acquired set of 800 images of which 300 are normal, 153 are stage 1 ROP, 239 are stage 2 ROP, 93 are stage 3 ROP, and 15 are stage 4 or above. DRIVE, STARE, CHASE_DB1 and HRF used for training | ROP-GAN synthesis model achieved higher classification scores by CNN models than other segmentation methods (91% for Resnet34 compared to 73.2% based on original images alone). |
| Table 3 (continued) | | | | | |
| Disease/  Study | **Year** | **Generative**  **aspect** | **Method** | **Datasets** | **Reported Results** |
| ROP/  Coyner et al. [73] | 2022 | Augmentation | Previously-trained CNN segmented CF images into grayscale retinal vessel maps (RVMs), used to train PGGANs to generated synthetic RVMs.  PGGAN obtained from publicly available github repository. One CNN trained on real RVMs, one trained on synthetic ones. CNN performance compared to diagnoses made by 8 experts on 100 real RVMs | 3477 real images from 534 patients | CNN trained on synthetic RVMs achieved a significantly greater AUC (0.97) than the CNN trained on real RVMs (0.934). The synthetic-trained CNN agreed nearly perfectly with the diagnoses of 8 experts, with a calculated Cohen’s kappa of 0.922 |
| Multiple: DR, AMD, myopia, cataract, HR  glaucoma/  Han et al. [107] | 2021 | Anomaly detection | GAN network similar to an automatic encoder: model is trained only on normal samples and establishes a latent vector space of the distribution of normal images. Anomaly scores are calculated for input images, determining whether the input is likely an anomaly | Training: EyePACS, Local01, AEHI, IDRiD. External testing: Local-2, ODIR-5K, Messidor-2, JSIEC1000. Total 64,351 normal fundus images and 26,148 with lesions | For individual diseases: DR: AUC ~0.89, accuracy 80.4%; Glaucoma: AUC ~0.92, accuracy ~84%; Cataract: AUC ~0.91, acc ~83%. AMD: AUC ~0.87, accuracy ~78% Hypertensive Retinopathy: AUC ~0.9, acc 82%. Myopia: AUC 0.96 accuracy~ 89% |
| Glaucoma/  Chaurasia et al. [70] | 2024 | Data augmentation | DCGAN used to generate 20,000 each healthy & glaucoma optic disc images. DL CNN model (vgg19_bn) trained to detect GON (glaucomatous optic neuropathy) using purely synthetic, mixed and purely real images | 17,060 (6874 glaucomatous and 10,186 healthy) fundus images from 20 different databases worldwide; validated on Drishti-GS dataset | DL trained purely on synthetic (generated data) data performed poorly - AUC 73%. Including real data in training improved DL (AUC 86.5%) to virtually match that of DL trained only on real data (AUC 87.5%). |
| Glaucoma/  He et al. [69] | 2023 | Image conversion | Optain camera CF images input to pix2pixHD GAN, with Topcon camera pair image targeted as the output. The Optain-translated-to-Topcon (GAN-synthetic Topcon) image output then analysed using glaucomatous optic neuropathy (GON) deep learning model, classifying image as low, medium or high risk. | Two sets of fundus images collected from patients: one with Topcon and other using Optain camera. Excluding 189 pairs for low image quality, left 421 patients and 725 Topcon-Optain pairs. 843 Topcon-Optain pairs collected as external dataset | DL classification model showed many false positives on Optain images from external dataset, and far fewer on GAN-synthetic Topcon images. Overall, DL performance on Topcon images as reference, compared with (i) GAN-translated images, showed better agreement (Cohen’s kappa 0.60), vs compared with (ii) original Optain images. |
| Table 3 (continued) | | | | | |
| Disease/  Study | **Year** | **Generative**  **aspect** | **Method** | **Datasets** | **Reported Results** |
| Glaucoma/  Sreejith Kumar et al. [68] | 2022 | Augmentation | 990 healthy eye images and 862 glaucomatous eyes used to train two separate progressively-grown GAN (PGGAN) models to generate either normal or glaucomatous OCT images. Two experts assessed the quality and reality of the generated images. Performance of DL classifiers (VGG11 architecture) trained on (i) real images vs (ii) trained on synthetic images. | Patients with glaucoma from 4 clinical trials & controls from Singapore Epidemiology of Eye Disease program 1144 healthy eye and 1016 glaucomatous; data from Carol Davila University of Medicine and Pharmacy, Bucharest up to 150 normal and 150 glaucomatous. | Experts had limited ability to determine authenticity (accuracies of 51.8% and 51.3% at identifying real images. Some of the DL models trained only on synthetic images achieved comparable AUCs and in some cases higher AUCs when classifying glaucoma than DL models trained only on real images - up to 0.95-0.97 vs 0.96-0.84 |
| Cataract/  Liu et al | 2024 | Image enhance | Incorporated spatial attention and multiscale discriminator as modifications to improve traditional CycleGAN, producing authors-termed C^2^ycleGAN. Pre-op and generated images evaluated in terms of quality, authenticity and diagnostic efficacy by 9 ophthalmologists, 3 each at resident, senior and expert level. | Unpublished from Zhongshan Ophthalmic Centre. 959 CF & 1009 UWF image pairs from 510 patients pre- and post-cataract surgery to train GAN; 100 CFP & 100 UWF pairs from 200 patients for testing | Clinical evaluation by ophthalmologists at resident, senior, expert levels showed average accuracies of retinopathy detection increased: 78% to 91% in CF images and 91% to: 93% in UWF images. GAN enhancement improved overall diagnostic accuracy for CF images from 57.3% to 73.7% for residents, 67% to 81% for seniors, and 69% to 81.7% in experts. For UWF images GAN enhancement showed significant increases from 62.0% to 73.7% for residents, 66% to 77% for seniors, and 71.7% to 79.7% for experts were observed. |
| Cataract/  Luo et al | 2020 | Image enhance  (de-haze) | Pre-trained U-Nets for segmentation of blood vessels (210 images total) and optic disc (500 images total) to evaluate de-haze performance.  400 unpaired clear and 400 cataractous images to train synthesis of 400 cataract/clear image pairs. Then pix2pix trained on synthesised image pair to dehaze 50 real cataract images & compare with matched images post-surgery | Data source for training images not specified; 50 real pre and matched post-surgery images from Eye Hospital of Wenzhou Medical University. | Compared with other dehaze algorithms, authors report overall improvements in BV segmentation vs best alternatives in sensitivity (0.744 vs 0.679), Dice 0.762 vs 0.646; correspondingly for OD authors report improved Dice (0.947 vs 0.928) and comparable or nearly so specificity for OD and BV. |
| Myopia/  Jiang et al. | 2019 | Improved segmentation | Conditional GAN based method including patchGAN discriminator, partial dense connections, Dice loss function and weighted binary cross-entropy loss for segmentation of linear lesions (mainly lacquer cracks and myopic stretch lesions) on ICGA images. Performance was evaluated by comparing segmentation results with ground truth. | Unpublished from Shanghai General Hospital: 152 ICGA images (two from each eye of 38 patients with linear lesions). Patients randomly allocated into four groups (10, 10, 9, 9) for four-fold cross validation. | Compared to other deep learning methods the proposed method achieves the best intersection over union ratio (52.95% vs 42.32% for best alternative), and best Dice similarity coefficient (69.21% vs 59.41%). |

Table Legend:

**Table 3** Summaries of papers describing Generative AI in diagnosis of ophthalmic disease, based on ophthalmic disease, generative aspect (e.g., augmentation, image conversion, anomaly detection, future image synthesis), datasets, and results.

Abbreviations and terms: BV – blood vessel; OD – optic disc; Dice – image similarity measure

**Table 4** Summaries of papers describing applications of GANs in Prognosis and Treatment of Ophthalmic Diseases

| Study | Year | Method | Dataset(s) | Results |
| --- | --- | --- | --- | --- |
|  |  |  |  |  |
| DME/  Baek et al. [78] | 2024 | Compare performance of GAN types: CycleGAN, UNIT, pix2pixHD and RegGAN on predicting anti-VEGF treatment at 52 weeks. Train with OCT at week 0 + infra-red fundus photos & retinal thickness vs train on OCT at 0, 4 and 12 weeks. | n = 327 (DME) eyes which underwent anti-vascular endothelial growth factor (VEGF) treatments every 4 weeks for 52 weeks; data from the randomized controlled trial (CRTH258B2305, KINGFISHER)  https://clinicaltrials.gov/study/NCT03917472 | CycleGAN, UNIT, Pix2PixHD, and RegGAN each generated 30 test OCT images, of which respectively 28, 29, 15, and 30 were gradable. RegGAN scored best in predictions of IRF, intraretinal fluid and hard exudates HE. |
| Glaucoma/  Hussain et al. [93] | 2023 | pix2pix GAN used to synthesise OCT B-scans at future time points and determine whether they help to improve prediction accuracy of change in visual field at 12 months. CNN model used to predict progression of disease: using real images | Data from a longitudinal study of patients undergoing trabeculectomy in Lithuania. 105 glaucomatous eyes (reduced to 86). OCT images, Visual field mean deviation, IOP values measured at five visits: baseline, months,3,6,9,12 (M3, M6, M9, M12). | Training CNN with real multimodal input until M6 combined with synthetic M9 and M12 images did not improve AUC significantly. But using real data only to M3 and adding synthetic M6, M9 and M12 images increased AUC to 0.81 (P = 0.038). |
| AMD (specifically neovascular AMD, nAMD)/  Zhao et al. [91] | 2023 | Developed Biomarkers-Aware Asymmetric Bibranch GAN. Dual branch GAN has adaptive memory batch normalisation (AMBN) in source branch & adaptive biomarkers-aware attention in target branch to enhance synthesis of local biomarker details. | GUM dataset: SD-OCT images from 4686 patients (65,320 used for training). Also self-generated dataset: 186 eyes from 186 patients with nAMD. All treated with 3 consecutive monthly injections of anti-VEGF. | Average prediction accuracy for the four biomarkers ranged from 81-92.1%. Comparing post-therapeutic images (real vs synthetic) gave model efficacy in predicting anti-VEGF treatment: accuracy 95.2% at 1 month, 95.2% at 3 months and 85.7% at 12 |
| AMD (specifically neovascular,  nAMD)/  Zhang et al.[108] | 2023 | Single-Horizon disease Evolution Network (SHENet) developed to predict post-therapeutic SD-OCT images: extends Pix2pix conditional GAN model. SHENet learns the progress of disease in the high-dimensional latent space | 46,208 paired SD-OCT images obtained from 22 nAMD patients (one eye per patient included). Images taken from around 17 different time points, each around 1 month apart when anti-VEGF injections were given | Quantitative evaluation: peak signal: noise ratio (PSNR), structural similarity (SSIM) and learned perceptual image patch similarity (LPIPS) were better than those achieved with the competing methods |
| AMD/  Moon et al. [90] | 2023 | Apply Attention-GAN to predict differences in short term outcome for patients treated respectively with anti-VEGF agents ranibizumab or aflibercept | 1684 OCT images from 842 patients: 419 patients treated with ranibizumab vs 423 with aflibercept.  Testing on 98 patients (49 each with ranibizumab and aflibercept; AI vs 2 retinal specialists | For ranibizumab group, AI scored in. sensitivity, specificity and accuracy respectively 0.615 0.667, 0.653 vs. corresponding averages for experts 0.308, 0.834, 0.694. For aflibercept group, AI scored 0.857 0.881 0.878 vs corresponding averages for experts of 0.429, 0.917, 0.847 |
| AMD/  Pham et al. [84] | 2022 | MuMO (multimodal) GAN based on Pix2pix to predict future drusen development as seen on CF images. CF image, drusen masks and time period are input; two discriminators are used to train generator to generate both realistic CF images and predict drusen development. | 8196 CF images from 1263 AMD patients acquired in a study at Kangbuk Samsung Hospital, Korea. 7156 images used for training, 1040 for testing | Results: drusen size of given image classified as non/small, intermediate or large. Comparing predictions between ground truth and synthesised images: average AUC of 0.65 and accuracy of 55% for 1, 3 and 5 years after baseline. |
| AMD/  Ganjdanesh et al. [80] | 2022 | cGAN takes paired images from two time points and generates future image based on input image alone (and time interval). CNN classifier (ResNet-18) scores image as advanced/not advanced AMD on 12-level scale. Binary and 3-class classifiers used to separate AMD onset times. | National Eye Institute AREDS (187,996 CF images from 4628 subjects, up to 13 years’ worth of follow-up visit data). UK biobank: 175,546 fundus images of 85,278 subjects from 2 visits — 300 images (200 control and 100 with advanced AMD) manually extracted as an independent test set. | GAN + binary classifier achieved accuracy on the test set of: 0.734 at 2 years, 0.7529 at 3 years, 0.766 at 4 years. GAN + 3 class classifier achieved accuracy of 0.7127 at 2 years, 0.7181 at 3 years, 0.6671 at 4 years |
| AMD (nAMD)/  Lee et al. [88] | 2021 | Pix2pix-based cGAN to synthesise post-treatment OCT images from input baseline images. Four GANs trained (OCT only, OCT with FA, OCT with ICGA or OCT with both FA and ICGA). 150 OCT images generated from the 150 baseline OCT images in the test set. Two ophthalmologists assessed the generated and real images. | Spectral domain OCT volume scans of 314 eyes in 303 patients taken at two time points (baseline & 1 month after final injection of the loading doses. Additionally fluorescence angiography (FA) and indocyanine green angiography (ICGA) images obtained 5 minutes after dye injection | Best performance achieved when trained on OCT with FA and ICGA. Sensitivity low for intraretinal and sub retinal fluid (IRF, SRF) (24-33%), compared to 74-88% for pigment epithelial detachment (PED) and subretinal hyper reflective material (SHRM)  Specificity was high for all lesions (>95% when trained with FA and ICGA. |
| AMD (nAMD)/  Liu Y. et al. [89] | 2020 | pix2pixHD GAN trained on pairs of pre- and post-anti-VEGF therapy images. GAN produces post-therapy images based on pre-therapy image. Two retinal specialists graded images for quality & realness, then assessed macular status - wet with either intraretinal and/or subretinal fluid, or dry, followed by whether the macula changed from wet to dry from pretherapeutic to real / synthetic post therapeutic. | Pre-therapeutic and post-therapeutic  B-scan swept-source OCT (SS-OCT) Topcon images taken from 526 patients (476 pairs assigned to the training set, 50 to testing) Retrospective data from Peking Union Medical College Hospital from November 2018 to June 2019. | 50 synthetic post-therapeutic images generated from the test pre-therapeutic image set, with 46/50 of enough quality for interpretation. Average specialists rate to discriminate real vs synthetic was 0.28. Macula status was ‘wet’ in 25/46 of the real post-therapy images. Specialists disagreed on one real post-therapeutic image, and on seven synthetics. Accuracy of synthetic OCT images to predict wet-to-dry conversion after a single dose of anti-VEGF was 0.81. |
| Thyroid eye disease/  Yoo et al. [103] | 2020 | Conditional GAN - here condition is orbital decompression status. GitHub links to DCGAN code and methods used for comparison (Pix2pix and CycleGAN) given. GAN trained on 76 pairs of training images, and generated 500 pairs of pre- and post-op images. DL classifier (VGG-16) trained either using only real training data, real data with simple augmentation techniques, or real data combined with the GAN-generated images. | No open database including both preoperative and matched postoperative face images. Matched preoperative and postoperative face photos from patients who underwent orbital decompression taken from Google Images, searching using related keywords. GAN model trained on 109 pre- and post-operative pairs (dataset was augmented slightly). Dataset split into 76 pairs for training a classifier and 33 for testing. | Synthetic post-op images generated by the DCGAN achieved better SSIM than those generated by Pix2pix and CycleGAN models, however the normalised MAE remained similar. The DL model trained on the 76 real pairs and the 500 GAN-synthesised pairs showed better performance on the test set (AUC 0.957) than either when trained only on real data (AUC 0.824) or on the real data with simple augmentation (AUC 0.872). DCGAN images had however low realism. |
| Blepharoptosis/  Sun et al. [43] | 2022 | Pix2Pix conditional GAN architecture trained on real pre- and post-op images, to generate eyelid structure predictions post-surgery. Four ophthalmology experts rated: predicted outcome of 75 pairs of pre-op and synthetic post-op images; and visual similarity of the 75 paired synthetic and real post-op images. | 970 pairs of pre- and post-op images from 450 eyes from 362 patients. 75 pairs of images used as a test set, remaining used for training and validation | Mean overlap ratio of predicted and real post-op images was 0.858. There were no significant differences in MPLDs between synthetic and real post-op eyelids at any angle. Ophthalmologists rated their overall satisfaction as “highly satisfied” in 420/750 cases and satisfied in 268/750 cases. |
| Cataract/  Frisch et al. | 2023 | MT-UNIT: Variational autoencoder-GAN. Motion Translator (MT) takes two consecutive frames + optical flow (OF) as input to UNet gives translated optical flow to translate motion in target domain, applying recycleGAN to ensure consistency between translated OF warped target image and predicted successor image. OF estimation uses pre-trained RAFT method | Open datasets Cataract101 (101 videos) and CATARACTS2020 (50 videos). From Cataracts2020, 25 videos used for training, 5 for validation and 20 for testing. From Cataract101 dataset, 70 videos used for training, 20 for validation, 11 for testing). | Results for authors MT-UNIT method compared with alternative methods to translate video from Cataract101 to CATARACTS2020 and vice versa (namely CycleGAN, RecycleGAN, UNIT, OF-UNIT.  MT-UNIT achieved better F1 score (0.285 vs 0.265 for OF-UNIT, the next best scoring model).  F1 = harmonic mean of precision P and recall R = (2xPxR)/(P+R) |
| Myopia/  Assaf et al. | 2024 | 5 million AS-OCT images generated by authors’ SWAGAN [99] were filtered by CNN to identify ICL scans and processed by image editing to generate a set of images with varying vault measurements. | Unpublished data. Post-op OCT scans from STAAR Surgical patients who underwent EVO or EVO+ ICL surgery. Training data: 2,447 AS-OCT B-scans from 70 eyes of 51 unique patients. Validation 2,110 images from 68 eyes of 52 patients. Testing 2,454 images of 88 eyes of 56 patients. | Addition of GAN synthetic data improved CNN performance, but relative effect much lower than image editing software. Filtered GAN generated synthetic data achieved an MAE of 35.10 µm for vault measurement, reducing to 24.83 µm when combined with vault resizing. |
